# It’s a Challenge, Not a Threat: Lecturers’ Satisfaction During the Covid-19 Summer Semester of 2020

**DOI:** 10.3389/fpsyg.2021.638898

**Published:** 2021-07-07

**Authors:** Martina Feldhammer-Kahr, Maria Tulis, Eline Leen-Thomele, Stefan Dreisiebner, Daniel Macher, Martin Arendasy, Manuela Paechter

**Affiliations:** ^1^Institute of Psychology, University of Graz, Graz, Austria; ^2^Department of Psychology, University of Salzburg, Salzburg, Austria; ^3^Department of Corporate Leadership and Entrepreneurship, University of Graz, Graz, Austria

**Keywords:** distance education, online instruction, challenge appraisal, lecturers’ satisfaction, teaching in higher education

## Abstract

The summer semester had just begun at Austrian and German universities when Covid-19 was declared a global pandemic by the World Health Organization. Thus, in March 2020, all universities closed their campuses, switching to distance learning within the span of about a single day. How did lecturers handle the situation? Were they still able to turn the situation into a positive one? What were the main obstacles with this difficult situation, and where there conditions which helped them to overcome the new challenges? These are research questions of the present survey with a sample of 1,152 lecturers at universities in Austria and Germany. The survey focuses on the lecturers’ appraisals of the novel situation as challenging or threatful. These appraisals are important for approaching a situation or shying away from it. However, how well a person adjusts to a novel situation is also influenced by personal and environmental resources which help to overcome the situation. The present survey focused on four possible sources of influence: internal assessments of the situation determining it to be threatening and/or challenging, personal resources, attitudes, and support by the organization. It was investigated to which degree these sources of influence could contribute to the lecturers’ satisfaction (or dissatisfaction) with their teaching processes. A multiple regression with three criterion variables describing university lecturers’ perceived satisfaction with distance teaching was carried out. Predictor variables were the lecturers’ appraisals of challenge and threat, perceived support by the university and sense of belonging to the university, temporal resources, proficiency in using digital technologies, length of teaching experience, and gender. Lecturers were mostly satisfied with their teaching activities. Together with the perception of a low threat potential, challenge appraisals contributed strongest to satisfaction. In comparison, assessments of actual personal resources, skills in the use of digital technologies, teaching experience, and temporal resources were important but contributed less to satisfaction than challenge appraisals. It seems that lecturers were only able to use these resources when the technological resources were available and when the lecturers were confident in their technical abilities.

## Introduction

The Covid-19 disease was identified for the first time at the end of 2019 in Wuhan, in the People’s Republic of China (Robert Koch Institute [RKI], 2020). The first confirmed Covid-19 case in the German-speaking realm was registered on January 27th, 2020 in Germany, and on February 25th in Austria. The summer semester had just begun at Austrian universities when on March 11th, Covid-19 was declared a global pandemic by the World Health Organization (WHO). To slow the spread of the virus, effective March 16th, extensive restrictions on many everyday activities were announced for all citizens by the Austrian federal government. In connection with these rapid developments, all universities closed their campuses, switching to distance learning within the span of about a single day (Kroisleitner, 2020; Neuhauser, 2020). In Germany, extensive travel restrictions were imposed on March 22nd. Here, universities decided in consultation with state and federal governments in mid-March 2020 to suspend all teaching until the end of the Easter break, which in most federal states was April 20th (German Rectors’ Conference, 2020). On April 15th, it was decided in Germany that teaching should resume in the form of distance (mostly online) formats, with the exception of specific on-campus courses where learning and teaching would not work well in a distance format (for example with laboratory work) and where appropriate safety measures were in place. In addition, all instructors in higher education in both countries now had to work and teach from home. With schools and daycare facilities closed, parents also had to simultaneously take care of their children. Several media reports suggest that these circumstances were particularly challenging for early-career researchers working toward their Ph.D. or tenure (Kramer, 2020; Pettit, 2020). Distance education was maintained in both Austria and Germany throughout the summer semester of 2020. Similar developments were seen in other countries worldwide, impacting over a billion learners (United Nations Educational and Scientific and Cultural Organization [UNESCO], 2020).

How did lecturers^1^ handle the situation? Were they still able to turn the situation into a positive one, and enjoy at least some satisfaction with their teaching? What were the main obstacles with this difficult situation, and which conditions facilitated their coping with their new challenges? These are the research questions of the present survey that examined lecturers at universities in Austria and Germany (two German-speaking countries with basically identical academic tertiary education systems).

## Variables That May Influence Lecturers’ Adjustment to Novel Teaching and Learning Conditions

How well a person adjusts to a novel situation like the one experienced in the summer semester of 2020 is influenced by the demands and goals a person sets for her/himself as well as by the personal and environmental resources they encounter in the workplace (Lazarus and Folkman, 1984; Uphill et al., 2019). In the present survey, we focused on four possible sources that might influence how lecturers successfully adjusted to the novel teaching and learning situation arising from the Covid-19 pandemic: internal assessments of the situation determining it to be threatening and/or challenging, personal resources, attitudes, and support by the organization.

*Appraisals of challenge or threat*. When individuals are faced with difficulties, they usually appraise what is at stake and what opportunities of action are available. So-called primary appraisal is concerned with the significance of a situation and the possible threat or challenge it poses to the individual. When an evaluation focuses on risks and factors that lie beyond the individual’s scope of action, the situation will be mainly perceived as a threat (Lazarus, 1999). In this case, individuals pay increased attention to the cues that signal their vulnerability and tend to withdraw from action (Wimmer et al., 2018). However, a new and difficult situation might also be perceived as a challenge or include elements of challenge offering opportunities for self-growth, gains in competences, satisfaction with the own performance, etc. When sizing something up as a challenge, individuals are more likely to shift to problem-solving, ultimately taking action to overcome adversity (Hase et al., 2019).

In comparison to primary appraisal, so-called secondary appraisal is concerned with the controllability of the situation. Secondary appraisal describes the degree to which an individual believes that she/he may control a difficult situation and influence the outcomes (Palmwood and McBride, 2019). Every situation has both threatening as well as challenging elements. They may however, differ in their degree of the challenges or threats prevailing within them (Seery, 2011). To what extent an individual experiences states of challenge and threat is strongly affected by one’s evaluation of personal resources and situational demands (Seery, 2011; Uphill et al., 2019). Here, different individuals may arrive at different appraisals of the same situation. It seems necessary to look not only at threat or challenge experienced by a person but also at the resources she/he disposes of.

*Lecturers’ personal resources and professional knowledge*. The Covid-19 pandemic in the summer semester of 2020 certainly could be understood as both threatening and challenging, with lecturers suddenly having to meet new demands in their teaching. A United States survey carried out in May 2020 with nearly 4,800 faculty members saw 91% of them report that they had to transition their courses to remote teaching; another 7% had already been teaching fully online and kept teaching in this form; while only 2% reported that their class had been canceled or suspended (Fox et al., 2020a, b). For many, the situation required the rapid acquisition of new knowledge and skills in the use of online technologies and instruction. In the survey by Fox et al. (2020a, b), more than half of the lecturers reported a lack of online teaching experience when the Covid-19 pandemic started. These lecturers were almost twice as likely to report that they struggled to adjust their teaching than their experienced colleagues; they not only now had to change to online instruction, but also had to acquire new knowledge and skills in distance (mostly online) teaching, all within a very short period of time. It remains unclear the degree to which experience with traditional face-to-face courses may help deal with new situations like this that arise. On the one hand, experienced lecturers might be at an advantage if they can look back upon extensive teaching expertise and knowledge in managing instructional tasks like course design, class management, learning assessment, etc. (Chang et al., 2011). On the other hand, they might view the use of a new technology more reluctantly, experiencing difficulties in transitioning from traditional didactic concepts and courses to new online formats (Li et al., 2018).

Generally, the task of moving face-to-face lessons online requires significant time and effort for each course. In addition, the process of teaching online is not less time-consuming than traditional in-person teaching (Chen, 2003; Wanner and Palmer, 2015). For most teaching staff, a variety of tasks ranging from classroom instruction, teaching preparation, grading, administrative work, committee meetings, grant-writing, research, publishing, etc. had to be reconciled to match new teaching tasks. Parents, especially women, faced additional difficulties with schools and daycare facilities being closed. Working at home suddenly had to be juggled with child care (Kramer, 2020). It’s therefore valid to assume that for many lecturers, the Covid-19 summer semester resulted in a higher work load and increased time demands. Female teaching staff might have been especially affected by teaching demands in this semester. Resources like time, professional experience, or external support may influence how well lecturers could manage the novel situation and its difficulties.

*Sense of belonging to an organization, support by the organization*. Obstacles at work feel easier to overcome if individuals identify with their organization. In these cases, they are more likely to perform their “work above and beyond their call of duty” (Mowday et al., 1982, p. 15; Wilkins et al., 2016) and show proactive behavior that protects or advances the organization. A sense of belonging forms an important dimension of organizational identification. It is defined as “the heavy identification with an organization, where the organization becomes part of their self-concept and the individual becomes psychologically connected to the organization in such a way that their own future is defined by the organization’s future” (Dávila and Jiménez García, 2012, p. 245). Sense of belonging and identification is also related to the support provided by an organization. The degree to which employees identify with their organization also depends on the extent to which they experience their organization valuing their contributions and caring about their well-being, e.g., by providing them with resources, information, showing appreciation, etc. (Marique et al., 2012; Strayhorn, 2019).

*Satisfaction (with one’s teaching).* The evaluation of the outcomes of one’s behavior describes not only an assessment of former achievement but also helps to identify future goals and areas of improvement. A positive evaluation and an attribution of success to own efforts and behaviors has implications for the future. A person who believes to dispose of the skills and resources to succeed in a situation will be likely to approach similar situations in the future and give her/his best (Doménech-Betoret et al., 2017; Weiner, 2019).

*Relationships between the variables.* From the viewpoint of learning and teaching in tertiary education, it is important to know how satisfied lecturers were with their teaching outcomes in the summer semester. Such a summative appraisal of performance outcomes influences future behaviors, allocation of individual resources, and assessment of a situation (Eccles and Wigfield, 2002). Behaviors to manage a novel situation depend on how the situation is assessed, as a challenge that can be approached, or a threat which might be rather avoided. Individual, internal as well as external resources (e.g., professional experience, time for preparing instruction, technical facilities provided) should have an influence on how lecturers are able to manage (and in a wider sense to control) the novel situation (Ohly, 2019), e.g., by spending more time for preparation, using additional resources. Resources are intertwined with the assessment of the situation as being challenging or threatful. To understand how lecturers perceived the novel situation due to the Covid-19 pandemic is important for preparing them for possible difficulties in the future.

Against this background, the present survey investigates via a large sample of lecturers at universities in Austria and Germany how well they managed the situation and which aspects contributed most to their satisfaction assessments.

## Materials and Methods

### Recruiting and Sample of Participants

From the beginning of June until the middle of July 2020, Austrian and German universities, universities of applied science, and colleges of teacher education were contacted by e-mail and asked to distribute information about the survey to their teaching staff (Feldhammer-Kahr et al., 2021). All of the recruited institutions offer educational programs on ISCED levels 5 and 6 (UNESCO, 2011). Altogether, 44 universities in Austria and 64 universities in Germany were contacted by e-mail and asked to forward information about the survey to their teaching staff. The survey was conducted online and programmed with LimeSurvey.

A total of 1,339 lecturers took part in the study. Due to missing values, the analyses presented in this paper are based on a smaller data set of 1,152 participants. The sample included 636 female (55.21%) and 516 male (44.79%) lecturers. Of the participants, 785 were from Austria (68.14%) and 367 from Germany (31.86%). These lecturers teach at different types of institutions [comprehensive universities with majors in sciences, arts, humanities, etc. and technical universities (*n* = 774, 67.2%), universities of applied sciences (*n* = 219, 19.0%), and colleges of teacher education (*n* = 159, 13.8%)].

The participants held different professional degrees and were contracted to a variety of employment forms. Some participants also were employed at more than one university. Therefore, in the following 1,152 participants having 1,197 kinds of employment are described: 98 (8.5%) professorships with higher managerial responsibility (e.g., dean, head of department); 153 (13.3%) professorships which included the responsibility for a research group; 160 (13.9%) professorships without these managerial functions; 234 (20.3%) positions as research and teaching assistants on track toward a doctoral or post-doctoral qualification; 150 (13.0%) positions as research and teaching assistants without the obligations described above; 158 (13.7%) positions as teaching assistants; and 244 (21.2%) positions as lecturers with a temporary contract (with the term professorship we refer to the Austrian and German employment laws, which usually encompass specific formal academic qualifications and obligations for teaching and research; see also European University Institute, 2021).

Following the classification of scientific disciplines by the Austrian Federal Ministry of Education, Science and Research (Bundesministerium Bildung and Wissenschaft und Forschung, 2020), the participants worked in the following fields: engineering (construction, architecture, computer science, electro technology) (*n* = 137, 11.9%); natural sciences and mathematics (including agricultural, forestry, psychology, etc.) (*n* = 249, 21.6%); medicine and public health care (including life science and veterinary medicine) (*n* = 64, 5.6%); humanities, social, educational and communication sciences (*n* = 126, 10.9%); law, business and economics, business education (*n* = 101, 8.8%); linguistics, cultural sciences, esthetics, music (*n* = 212, 18.4%); educational sciences and technical methodology (*n* = 238, 20.6%); and theology, religious studies, and philosophy (*n* = 22; 1.9%; three missing values in the data set, 0.3%).

To reduce concerns about the traceability of participants, we did not ask for the participants’ age. Participation was entirely voluntary and in accordance with the ethical standards of the institutional research committee. Informed consent was obtained from all participants of the study.

### Measures

*Satisfaction with teaching in the summer semester of 2020.* Three items which had been used in previous studies on satisfaction in online-learning were employed (Paechter et al., 2010; Luttenberger et al., 2018). Participants estimated the degree to which they could successfully and meaningfully implement their instructional concepts online (successful implementation) and the degree to which they could expand their own knowledge and skills (knowledge gains) in the 2020 summer semester. They furthermore assessed their overall satisfaction with teaching in the summer semester. Assessments were made on a five-point scale ranging from 1 (low satisfaction) to 5 (high satisfaction).

*Appraisal of challenge and threat*. The challenge-threat scale by Drach-Zahavy and Erez (2002), which shows high reliability coefficients, was employed. The instrument measures challenge-threat appraisals in various work contexts. Eight items of the scale were employed; they had to be changed slightly for use in universities. For use in the present study, the instrument was translated from English to German and back translated. In the present sample, the structure of the questionnaire was investigated by means of principal axis factor analysis with orthogonal rotation (varimax with Kaiser normalization) in the present data set. Two factors were identified: Threat appraisals were measured by five items describing the respondents’ perceptions of the expected consequences of the situation for them. Challenge appraisals were measured by three items describing the lecturers’ overall expectations of success. Reliability indices measured by Cronbach’s α were satisfactory, with α = 0.813 for the threat and α = 0.823 for the challenge scale. Item examples included: “The situation seemed like a threat for me.” or “The situation provided opportunities to overcome obstacles.” (original items used the word “task” instead of situation). Ratings were made on a five-point scale ranging from 1 (not true) to 5 (true). Thus, high values indicated high threat appraisal and high challenge appraisal, respectively.

*Lecturers’ resources*. Teaching experience was measured by the item “How long have you been teaching at the university?” Answers here were categorized ranging from 1 (less than one year) to 5 (16 years or more); the scaling was chosen in accordance to previous studies (Chang et al., 2011). Furthermore, lecturers assessed their proficiency in using digital technology by one item on a scale ranging from 1 (not proficient at all) to 5 (highly proficient). Lecturers then assessed their temporal resources, i.e., time they have available for preparing and conducting lectures (mostly working in the home office). Assessments ranged from 1 (very few) to 5 (very high).

*Sense of belonging and perceived support by the university.* A questionnaire on the sense of belonging/identification with and support received by the university was developed based on existing measures (Zaussinger et al., 2016; Arslan and Duru, 2017; Organisation for Economic Co-operation and Development [OECD], 2017; Marksteiner et al., 2019). Its structure was investigated by means of principal axis factor analysis with orthogonal rotation (varimax with Kaiser normalization) in the present data set. It yielded two factors with five items measuring belonging/identification (example item: “I feel appreciated by my institution”) and two items measuring support (“I receive technical support by my institution,” “I receive support concerning didactical issues”). Cronbach’s α were satisfactory with α = 0.823 for belonging and α = 0.800 for support. Ratings on a five-point scale ranged from 1 (not true) to 5 (true). Here, high values indicated more positive assessments.

The study was performed in accordance with the American Psychological Association’s Ethics Code and the Declaration of Helsinki. It was carried out within a larger project on learning and instruction which had been approved by the University of Salzburg. All participants gave their written informed consent to participate, and to confirm that their data were able to be used in an empirical study. The questionnaire can be found in the appendix. For regression analyses the software lavaan (Rosseel, 2012) was employed.

## Results

Descriptive statistics for variables are shown in Table 1. Correlations between variables are shown in Table 2.

**TABLE 1:** Descriptive statistics (*M*, *SD*, *MD*, *IRQ*, *min*, *max*), skewness, kurtosis.

	***M***	***SD***	***Md***	***IRQ***	***min***	***max***	**skewness**	**kurtosis**
Overall satisfaction	3.52	1.18	4.00	1.00	1.0	5.0	−0.52	−0.52
Successful implementation	4.00	1.00	4.00	2.00	1.0	5.0	−0.95	0.62
Knowledge gains	4.06	1.00	4.00	1.00	1.0	5.0	−1.08	0.88
Threat	1.60	0.70	1.40	1.00	1.0	4.8	1.40	1.91
Challenge	3.40	1.04	3.33	1.67	1.0	5.0	−0.32	−0.56
Sense of belonging	3.97	0.82	4.20	1.20	1.4	5.0	−0.72	−0.09
Perceived support	3.56	1.08	3.50	1.50	1.0	5.0	−0.41	−0.60
Temporal resources	3.48	1.16	4.00	1.00	1.0	5.0	−0.40	−0.62
Proficiency in digital technologies	4.05	0.81	4.00	1.00	1.0	5.0	−0.52	−0.12

*Scale range from 1 (low satisfaction, challenge, threat etc.) to 5 (high). *M*, mean; *SD*, standard deviation; *MD*, median; IRQ, interquartile range; *min*, minimum; *max*, maximum. Sample size for all variables *n* = 1,152.*

**TABLE 2:** Correlations between variables (Spearman-Rho above, Pearson below primary diagonal).

	**1**	**2**	**3**	**4**	**5**	**6**	**7**	**8**	**9**
1 Overall satisfaction		0.587**	0.379**	−0.354**	0.478**	0.207**	0.261**	0.314**	0.209**
2 Successful implementation	0.602**		0.317**	−0.332**	0.396**	0.159**	0.178**	0.201**	0.297**
3 Knowledge gains	0.394**	0.352**		−0.035	0.648**	0.179**	0.167**	0.083**	−0.001
4 Threat	−0.339**	−0.316**	−0.031		−0.046	−0.195**	−0.127**	−0.212**	−0.325**
5 Challenge	0.490**	0.423**	0.682**	−0.055		0.152**	0.191**	0.120**	0.031
6 Sense of belonging	0.214**	0.160**	0.168**	−0.181**	0.156**		0.496**	0.147**	0.047
7 Perceived support	0.278**	0.201**	0.168**	−0.140**	0.194**	0.490**		0.208**	0.037
8 Temporal resources	0.311**	0.205**	0.087**	−0.216**	0.129**	0.153**	0.229**		0.068*
9 Proficiency in digital technologies	0.220**	0.307**	0.020	−0.329**	0.048	0.053	0.062*	0.071*	

*p < 0.05; **p < 0.01.

For the length of teaching experience, the median was *Md* = 3 (six to ten years teaching experience); 74 (6.4%) of the participants had less than one year teaching experience, 263 (22.8%) one to five years, 245 (21.3%) six to ten years, 187 (16.2%) eleven to fifteen years, and 383 (33.2%) more than 16 years teaching experience (*n* = 1,152).

A multiple regression with three criterion variables was carried out to investigate which aspects contributed to lecturers’ perceived satisfaction with distance teaching in the summer semester of 2020 (overall satisfaction, successful implementation of the instructional concept, knowledge gains in the summer semester). Predictor variables were the lecturers’ appraisals of challenge and threat, their sense of belonging and perceived support of the university, temporal resources, proficiency in using digital technologies, and the length of teaching experience. Furthermore, gender was included as a predictor variable (the multiple regression analysis summary can be found in Table 3). We assumed a linear relation between independent (predictor) and dependent (criterion) variables, meaning we would expect that increases in one variable would be related to increases or decreases in another. lavaan software (Rosseel, 2012) was used for the multiple regression analyses because it supports the investigation of the relationship between a set of independent and dependent variables in one single regression. When interpreting results, it’s important to note that multiple regression does not explain causes and effects, but instead describes the relations between variables or sets of variables.

**TABLE 3:** Summary of the multiple regression analysis (regression coefficients, significance).

	**Overall satisfaction**				**Successful implementation of teaching concept**				**Knowledge gains**			
	***B***	***SE B***	**β**	***Sign.***	***B***	***SE B***	**β**	***Sign.***	***B***	***SE B***	**β**	***Sign***
Threat	−0.482	0.050	−0.248	***	−0.376	0.052	−0.208	***	0.032	0.055	0.015	
Challenge	0.563	0.031	0.436	***	0.472	0.033	0.392	***	0.980	0.041	0.704	***
Sense of belonging	0.052	0.044	0.032		0.028	0.047	0.018		0.121	0.051	0.069	*
Perceived support	0.127	0.034	0.102	***	0.065	0.038	0.056		0.021	0.038	0.016	
Temporal resources	0.220	0.028	0.190	***	0.099	0.031	0.092	**	−0.012	0.031	−0.009	
Teaching experience	−0.063	0.026	−0.063	*	−0.006	0.027	−0.006		0.057	0.027	0.053	*
Proficiency in digital technologies	0.180	0.044	0.109	***	0.364	0.047	0.237	***	0.002	0.044	0.001	
Gender	−0.053	0.071	−0.020		−0.072	0.073	−0.029		−0.059	0.075	−0.020	
*R*^2^	0.445				0.360				0.521			

B, unstandardized regression coefficient β; SE B, standard error of B; β, standardized regression coefficient. *p < 0.05; **p < 0.01; ***p < 0.001. Sample size n = 1,152.

Six variables significantly contributed to the overall satisfaction with one’s teaching in the summer semester of 2020. Four variables obtained a positive β-weight: appraisal of challenge, temporal resources, proficiency in digital technologies, and perceived support by the organization (variables ordered according to their weight). Two variables were negatively associated with satisfaction: for appraisal of threat and length of teaching experience, higher values were related to decreased satisfaction. A high amount of variance (44.5%) could be explained by the regression equation.

Four variables significantly contributed to the assessment of whether lecturers could successfully implement their teaching concept; three of them obtained a positive β-weight: appraisal of challenge, proficiency in digital technologies, and temporal resources (variables ordered according to their weight). Appraisal of threat was negatively associated with the lecturers’ assessment that they were able to implement their teaching concept well. Altogether, 36% of the variance could be explained by the regression equation.

Three variables significantly contributed to the lecturers’ perceived knowledge gains: appraisal of challenge, sense of belonging, and length of teaching experience. A large amount of variance (52.1%) could be explained by the regression equation.

## Discussion

The present survey provides insights into lecturers’ experiences in the Covid-19 pandemic during the 2020 summer semester as well as into the development of respective support measures to assist their transition from in-person to online teaching.

### Sources of Satisfaction

Three aspects of lecturers’ satisfaction were recorded in the survey: overall satisfaction, lecturers’ satisfaction with the implementation of their instructional concept, and perceived knowledge gains.

*Satisfaction with teaching during the Covid-19 summer semester*. The mean and median of lecturers’ satisfaction with their teaching activities (both above the scale mean of 3) indicates that the participants did in fact obtain a sense of satisfaction from the teaching situation. The situation was regarded as a challenge and had a lower potential of threat (mean and median below the scale mean of 3). It seems plausible that lecturers maintained a positive view of their teaching situation during the 2020 summer semester. The scale for challenge (Drach-Zahavy and Erez, 2002) with items expressing appraisal of the situation as an opportunity to gain knowledge and overcome barriers contributed strongly to satisfaction. As a corollary, perceived threat impaired satisfaction with the situation. The assessment of the situation as a challenge might be explained by the lecturers’ positive assessment of their temporal resources, their proficiency in using digital technologies, as well as by the support of their organization, even though these variables received a lower weight in the regression equation. Challenge appraisals take an assessment of resources into consideration. However, they can be regarded as immediate assessments and important factors for the decision to approach a situation and to invest effort (Hase et al., 2019). Therefore, it is plausible that challenge appraisals received a higher weight in the regression analyses. Longer teaching experience was somewhat surprisingly related to lower satisfaction. It might have been that these lecturers had a wider range of experiences and best-practice examples for teaching which served as a frame of reference for their more negative evaluation. Gender contributed to none of the satisfaction measurements.

*Successful implementation of one’s teaching concept*. The mean and median of 4.00 for the items indicates that the participants were mostly satisfied with how they implemented their online teaching concept. Again, the perception of the situation as a challenge strongly contributed to satisfaction with the own teaching. Appraisal of the situation as a threat or challenge were the strongest contributors in the regression analysis. Stronger appraisal of the situation as a threat, a focus on one’s own deficiencies, and lack of knowledge diminished satisfaction. Not surprisingly, proficiency in use of digital technologies, in combination with sufficient time for moving instruction to an online format, and carrying out distance teaching contributed positively to satisfaction.

*Knowledge gains in the teaching situation.* The mean value of 4.06 (median 4.0) for the item indicates a positive assessment of individual knowledge gains in the Covid-19 summer semester of 2020. Appraisal of the Covid-19 situation as a challenge was again the strongest predictor of lecturers’ positive assessment. However, a sense of belonging also positively contributed, and probably served as a motivator for learning (Stoll et al., 2006). Somewhat surprisingly, longer teaching experience was positively related to higher perceptions of knowledge gains. Lecturers with longer experience and who were older in age probably already had well-established traditional teaching concepts in place, and needed to overcome more obstacles to transition their courses online; thus, they might have experienced a wide range of chances for knowledge gains. Or from the viewpoint of younger lecturers: it could be that they had already taught online, or at least were more eager to embrace online technologies, and could rely on their existing knowledge, whereas older lecturers had to obtain new knowledge and skill sets (see also Wingo et al., 2017).

### The Role of Challenge and Threat Appraisals in Comparison to Personal and Organizational Resources

Looking closer at the job characteristics of lecturers can help provide greater insight into this study’s results. University lecturers typically enjoy high responsibility along with high autonomy in their work (Sharma and Jyoti, 2009). The activity of teaching itself, e.g., being able to establish a relationship with students is also mostly regarded as a satisfying element of the profession (Szromek and Wolniak, 2020), constituting a source of social support, something seen as an important job satisfaction variable (Gerich and Weber, 2020). Even in a novel and difficult situation like the one experienced during the summer semester of 2020, there were still certain degrees of freedom for implementing new instructional concepts.

The results indicate that the lecturers were mostly satisfied with their teaching activities, and felt they were able to overcome the barriers presented by the situation. Altogether, they emphasized the merits of challenge appraisals. Together with the perception of a low threat potential, challenge appraisals contributed strongest to satisfaction. Appraising the teaching situation as a challenge was related to a focus on the opportunity for self-growth and gains in knowledge and skills (when considering single item formulations). These kinds of challenge appraisals are usually associated with more positive expectations for success (Skinner and Brewer, 2002).

In comparison, assessments of actual personal resources, skills in the use of digital technologies, teaching experience, and temporal resources were important but contributed less to satisfaction than challenge appraisals. It should be noted that the participants regarded their temporal resources as well as their skills and knowledge in the use of digital technologies rather favorably and as sufficient for their teaching. With this in mind, the mere disposal of resources appeared insufficient for handling difficulties experienced in the Covid-19 semester successfully, and needed to be accompanied by confidence in the own ability to overcome barriers, along with an attitude that the situation would also offer opportunities for self-growth and knowledge gains.

### Implications for Universities

The above results are important for how universities can support their teaching staff in difficult situations like the one encountered in the summer semester of 2020.

Technical and didactic support contributed to the lecturers’ overall satisfaction as well as to their assessment of a successful transition to online teaching. This support can be provided in various forms. Many universities expanded their digital learning infrastructure by upgrading existing software tools, providing new software and hardware, upgrading hardware infrastructure, or rolling out new collaboration and video conferencing software. Didactic support included measures like counseling from discipline-specific experts, providing didactic information via newsletters, *ad hoc* web courses, peer review, etc.^2^

A qualitative analysis of the participants’ additional responses to open questions about their individual needs for support by their institution (Tulis et al., 2021) confirmed the suggestions above. More than the half of the participants used this optional possibility to express the kind of support they would require or find helpful from their university. Most of them indeed focused on technical support (e.g., workshops, IT support, equipment, and software tools) but also on the provision of concepts for online teaching and online learning assessments by their university. Because threat appraisals are partially based on the experience of having insufficient resources to meet situational demands (Blascovich and Mendes, 2000), this kind of support may enhance lecturers’ perceived resources.

Temporal resources for planning, preparing, and implementing online teaching, and working from home during the Covid-19 semester were also significant predictors of satisfaction. Institutions can support lecturers’ limited time in various ways, e.g., by providing support from technical staff for moving instruction online, in the form of support in case of problems, or by providing good-practice examples including various learning scenarios that can be exported and adapted by lecturers. Further measures could include fostering production and collaborative re-use of teaching materials, e.g., by providing access to Open Educational Resource (OER).^3^

Lecturers’ proficiency in using digital technologies was also a significant predictor of satisfaction and the positive evaluation of one’s teaching. This finding points to the importance of training measures for teaching staff. Long-term, lecturers’ self-assessments are strongly related to the universities’ didactic support of their teaching staff, along with their human resource development. This might include training programs, on-the-job training, qualification opportunities for all career stages, and individual measures like coaching and mediation^4^.

The results of the survey emphasize lecturers’ assessments of the situation as a challenge as an important contribution to satisfaction; these assessments are connected to personal resources and attitudes. Appraisal of the situation as a challenge contributed positively to satisfaction. Different explanations are possible; one might be that the lecturers received sufficient support, that they felt they were able to overcome difficulties of the new situation.

Altogether, the results advocate not one or a few specific technological, organizational, or other measures, but speak in favor of bundles of measures that can be tailored to individual lecturers’ needs. From an organizational view point it would be also interesting and important to identify groups of lecturers with similar needs so that courses, support, or services could be administered to a group instead of a single person.

Universities already offer their teaching staff larger degrees of freedom for fulfilling their complex tasks. These degrees of freedom should of course be maintained, and support measures should take individual needs and resources into consideration. This would for example mean not proposing a single technical solution or a sole didactic concept for an entire organization, but instead providing different solutions which fit individual demands or the needs of specific groups (e.g., teaching staff in a specific major). Although universities cannot offer an unlimited range of technological tools or support, within a certain framework, it should nevertheless be possible to consider individual needs and resources. The results of this survey recommend considering lecturers’ very individual assessments of teaching situations, providing them with customized support measures, and raising their proficiency in an effort to hopefully achieve assessments from them that understand situations like the Covid-19 2020 summer semester as challenging but manageable.

## Limitations and Outlook

Partly, limitations of this study concern the design of the survey. It has to be kept in mind that the cross-sectional design poses limitations on the causal interpretation of data; on the other hand, the design made it possible to investigate a large group of participants. Other limitations concern the voluntary self-recruitment of participants (Fricker, 2012). Tertiary higher education institutions were contacted by e-mail for this study and asked to distribute the online questionnaire among their teaching staff. The research team had an influence on neither the universities’ nor the lecturers’ willingness to contribute. This kind of selection bias is nearly unavoidable in surveys with voluntary participation. Descriptive statistics point at the participants’ high satisfaction with the teaching situation in the summer semester of 2020. Perhaps a self-selection of lecturers having more positive attitudes occurred. However, even in this (self-)selected group of participants, challenge appraisals and a positive outlook on the situation were important. If this was in fact the case, this means it will be even more important to strengthen lecturers via a more critical assessment of resources, supporting them in experiencing this kind of difficult situation as a challenge and an opportunity to learn.

Altogether, the results point at variables that are able to explain satisfaction; interestingly an appraisal of the novel situation as challenging resulted in higher satisfaction. The results deliver valuable information for institutions in higher education about how to perceive and support their teaching staff. They also point at measures of support. By providing a broad range of services and resources tailored to individual needs, universities can help their teaching staff overcome difficulties. Considering that lecturers before the Covid-19 crisis were found to be reluctant to do distance teaching, with the lack of institutional support and time commitment being the main concerns (Wanner and Palmer, 2015; Feldhammer-Kahr et al., 2019), the Covid-19 situation delivered opportunities to expand existing support measures and thus foster teachers’ confidence and the future use of distance learning.

The present survey was carried out in an extraordinary situation which could not have been foreseen by anyone and which demanded a lot from educators. The results provide a snapshot and can be understood as a description of how lecturers assessed and handled the first Covid-19 semester. At the present time universities face their third semester of online teaching. For a deeper understanding it would be important and interesting to carry out further surveys and to know more whether and how lecturers adapted to the situation and whether their assessments of and their engagement in the situation changed.

## Data Availability Statement

The raw data supporting the conclusions of this article will be made available by the authors, without undue reservation.

## Author Contributions

MF-K, MT, EL-T, SD, DM, MA, and MP made a substantial, direct and intellectual contribution to the work, and approved it for publication. All authors contributed to the article and approved the submitted version.

## Conflict of Interest

The authors declare that the research was conducted in the absence of any commercial or financial relationships that could be construed as a potential conflict of interest.

## Footnotes

1 The term Universitätslehrende (translation university lecturers) used in the German-speaking realm for teaching staff at universities refers only to the activity of teaching and does not imply any specific contract or employment status. We will use the term “lecturer” in the following.

2 See https://www.uni-hildesheim.de/e-learning2020/

3 for example, www.oernds.de/

4 for example, personalwesen.univie.ac.at/en/services-for-employees/human-resources-development/; personalressort.uni-graz.at/de/abteilungen/personal-und-organisationsentwicklung/
