# Supplementary material for: It’s a Challenge, Not a Threat: Lecturers’ Satisfaction During the Covid-19 Summer Semester of 2020
Source: Front Psychol. 2021 Jul 7;12:638898. doi: 10.3389/fpsyg.2021.638898 (PMC8292608; doi:10.3389/fpsyg.2021.638898)
Supplement: Supplementary file 1 [file Data_Sheet_1.pdf]

Martina Feldhammer-Kahr[1], Maria Tulis[2], Eline Leen-Thomele[2], Stefan Dreisiebner[3], Daniel Macher[1], Martin Arendasy[1] and Manuela Paechter[1]*

[1] Institute of Psychology, University of Graz, Graz, Austria, [2] Department of Psychology, University of Salzburg, Salzburg, Austria, [3] Department of Corporate Leadership and Entrepreneurship, University of Graz, Graz, Austria

*Correspondence:
Manuela Paechter
manuela.paechter@uni-graz.at

Questionnaire on Challenge/Threat/Satisfaction in Teaching in Higher Education (German Version)

Supplementary Material to the Article by

Feldhammer-Kahr M, Tulis M, Leen-Thomele E, Dreisiebner S, Macher D, Arendasy M and Paechter M (2021) It’s a Challenge, Not a Threat: Lecturers’ Satisfaction During the Covid-19 Summer Semester of 2020. Front. Psychol. 12:638898. doi: 10.3389/fpsyg.2021.638898

Fragebogen

TeilnehmerInneninformationen und Einwilligungserklärung

Sehr geehrte Lehrende,

vielen Dank für Ihr Interesse an dieser wissenschaftlichen Befragung. Bevor Sie an dieser Studie teilnehmen können, benötigen wir von Ihnen eine Einverständniserklärung. Dazu einige Informationen zur Studie:

Allgemeine Informationen

Diese Erhebung erfolgt im Rahmen des BMBWF geförderten Forschungsprojekts "On track" der Universität Salzburg in Kooperation mit der Universität Graz und stellt eine Befragung von Lehrenden im Sommersemester 2020 dar.

Aufgrund der COVID-19 Eindämmungsmaßnahmen ist die Lehre an Hochschulen in diesem Semester weitgehend digital gestaltet. Diese Befragung dient dem Zweck, die Veränderungen der aktuellen Lehrsituation und deren Auswirkungen auf die Lehre zu erfassen, um Empfehlungen und Unterstützungsmaßnahmen für die Zukunft ableiten zu können.

Ablauf

In dieser Studie werden zunächst einige Informationen zu Ihren demografischen Daten und Ihrer beruflichen Tätigkeit erhoben. Im Anschluss daran werden Sie zu Ihren aktuellen Lehrveranstaltungen, Ihren Rahmenbedingungen und verfügbaren Ressourcen sowie Ihren Einschätzungen der online-Lehre in diesem Semester befragt.

Die Bearbeitung dieser Studie wird ca. 15 Minuten in Anspruch nehmen.

Ausschlusskriterien und Rechte

Für diese Studie liegen keine Ausschlusskriterien vor. Ihre Teilnahme an der Studie erfolgt freiwillig und sie können sich jederzeit, auch ohne Angabe von Gründen, von der Studie zurückziehen und/oder eine Löschung Ihrer Daten beantragen, ohne dass Ihnen daraus Nachteile irgendwelcher Art entstehen.

Pflichten

Um einen reibungslosen Ablauf der Studie zu gewährleisten, sind wir auf Ihre Mitarbeit angewiesen. Das bedeutet, dass es sehr wichtig ist, dass Sie den vorgesehenen Ablauf einhalten, die Instruktionen genau lesen und wahrheitsgetreu bzw. nach bestem Wissen und Gewissen antworten. Nur so können sinnvolle Empfehlungen und praxisrelevante Erkenntnisse aus den Daten gewonnen werden.

Nutzen

Ihre Teilnahme hat wahrscheinlich keinen persönlichen Nutzen für Sie selbst. Die gewonnenen wissenschaftlichen Erkenntnisse aus dieser Studie tragen dazu bei, Auswirkungen der digitalen Lehre

und damit verbundene Bedürfnisse von Lehrenden zu erfassen. Nach Studienende erhalten Sie auf Wunsch Informationen zu den Ergebnissen der Studie.

Risiken und Unannehmlichkeiten

Die beschriebenen Prozeduren sind nicht gesundheitsschädlich und entsprechen internationalen wissenschaftlichen Standards. Vom oben dargestellten Ablauf und den Messprozeduren gehen jedoch einige geringe Risiken bzw. Unannehmlichkeiten aus: Unter Umständen können Sie vorübergehend negative emotionale Empfindungen hervorrufen. Diese Empfindungen sowie spätere diesbezügliche Erinnerungen sind jedoch vorübergehender Natur.

**Welchem Geschlecht fühlen Sie sich zugehörig?**		
❑ weiblich	❑ männlich	❑ divers

**An welcher Institution lehren Sie?**		
❑ Universität / Technische Universität	❑ Fachhochschule	❑ Pädagogische Hochschule

**In welchem Land befindet sich diese Institution?**			
❑ Österreich	❑ Deutschland	❑ Schweiz	❑Luxembourg
❑ Sonstiges, nämlich: ________________________			

**Wie lange lehren bzw. unterrichten Sie bereits an einer Hochschule/ Universität?**			
❑ Weniger als 1 Jahr	❑ 1 bis 5 Jahre	❑ 6 bis 10 Jahre	❑ 11 bis 15 Jahre
❑ 16 Jahre und mehr			

**Welchen Tätigkeitsbereich/ welche Funktion erfüllen Sie an Ihrer Universität/ Hochschule?**			
❑ Professur mit Leitungsfunktion über eine größere organisatorische Einheit (Dekanat, Institut etc.)	❑ Professur mit Arbeits-bereichsleitung	❑ Professur ohne/mit geringer Leitungsfunktion (z.B. Juniorprofessur, Assoziierte Professur)	❑ Wissenschaftliche/r Mitarbeiter/in in der Qualifizierungsphase (z.B. Dissertation, Habilitation, ...)
❑ Wissenschaftliche Mitarbeiter/in ohne Qualifizierungsvereinbarung	❑ Stelle, die haupt-sächlich Lehre umfasst, z.B. Lecturer-Stelle	❑ Lehrbeauftragte/r	❑ Sonstiges, nämlich: _____

**Wie sicher fühlen Sie sich generell im Umgang mit digitalen Technologien?**	1 – Gar nicht sicher	2 – Eher un-sicher	3 – Teils/ teils	4 – Eher sicher	5 – Sehr sicher
	①	②	③	④	⑤

**Wie gut sind Sie in Bezug auf zeitliche Ressourcen für die Vorbereitung und Durchführung der Lehre von zuhause aus ausgestattet?**	1 – Sehr schlecht	2 – Eher schl-echt	3 – Teils/ teils	4 – Eher gut	5 – Sehr gut
	①	②	③	④	⑤

**Ein paar Fragen zu Ihren Erfahrungen mit der digitalen Lehre in diesem (Corona-) Semester.**	1 – Stimmt nicht	2 – Stimmt eher nicht	3 – Teils /teils	4 – Stimmt eher	5 – Stimmt
Im Umgang mit den digitalen Lehrangeboten (z.B. Lernplattform unserer Hochschule) fühlte ich mich sicher.	①	②	③	④	⑤
Ich konnte meine Lehre sinnvoll digital umsetzen.	①	②	③	④	⑤

**In der aktuellen Lehrsituation in <u>diesem</u> Semester ...**	1 – Stimmt nicht	2 – Stimmt eher nicht	3 – Teils /teils	4 – Stimmt eher	5 – Stimmt
... konnte ich viel Neues lernen.	①	②	③	④	⑤
... war ich im Allgemeinen zufrieden.	①	②	③	④	⑤

**Wie sehr stimmen Sie folgenden Aussagen zu, wenn Sie an die aktuelle Situation denken?**<br>*Quelle: adaptiert von Drach-Zahavy and Erez (2002)*	1 – Stimmt nicht	2 – Stimmt eher nicht	3 – Teils /teils	4 – Stimmt eher	5 – Stimmt
Die Situation gab mir die Möglichkeit, meine Fähigkeiten zu erweitern.	①	②	③	④	⑤
Die Situation bot mir die Möglichkeit, Hindernisse zu überwinden.	①	②	③	④	⑤
Die Situation stellte für mich eine Möglichkeit dar, um mein Selbstwertgefühl zu stärken.	①	②	③	④	⑤
Die Situation stellte für mich eine Bedrohung dar.	①	②	③	④	⑤
Ich machte mir Sorgen, dass die Situation meine Schwächen aufzeigen könnte.	①	②	③	④	⑤
Im Großen und Ganzen erschien es mir so, als könnte ich die Situation nicht meistern.	①	②	③	④	⑤
Ich machte mir Sorgen, dass es mir an Fähigkeiten mangelt, die Situation zu meistern.	①	②	③	④	⑤
Im Allgemeinen dachte ich, dass ich die Situation meistern kann.	①	②	③	④	⑤

**Wahrgenommene Unterstützung**<br>*Quelle: adaptiert von Arslan & Duru (2017); Marksteiner et al. (2019); Organisation for Economic Co-operation and Development [OECD] (2017); Zausinger et al. (2016)*	1 – Stimmt nicht	2 – Stimmt eher nicht	3 – Teil s/tei ls	4 – Stimmt eher	5 – Stimmt
Ich kann mich mit meiner Hochschule gut identifizieren.	①	②	③	④	⑤
Ich fühle mich in meiner Hochschule wertgeschätzt.	①	②	③	④	⑤
Ich fühle mich in meiner Hochschule didaktisch unterstützt (z.B. durch Bereitstellung von Informationen)	①	②	③	④	⑤
Ich fühle mich in meiner Hochschule technisch unterstützt (z.B. durch Bereitstellung von Informationen)	①	②	③	④	⑤
Ich habe das Gefühl, an meiner Hochschule nicht richtig dazuzugehören.	①	②	③	④	⑤

Ich bin gerne als Lehrender an meiner Hochschule tätig.	①	②	③	④	⑤
Ich habe viele Kontakte zu den Lehrenden in meiner Hochschule/an meinem Fachbereich.	①	②	③	④	⑤
Ich fühle mich sozial gut in meiner Hochschule eingebunden.	①	②	③	④	⑤

Literature

Arslan, G., and Duru, E. (2017). Initial development and validation of the school belongingness scale. *Child Indic. Res. 10*, 1043–1058. doi: 10.1007/s12187-016-9414

Drach-Zahavy, A., and Erez, M. (2002). Challenge versus threat effects on the goal-performance relationship. *Organ. Behav. Hum. Decis. Process. 88*, 667–682. doi: 10.1016/S0749-5978(02)00004-3

Marksteiner, T., Janke, S., and Dickhäuser, O. (2019). Effects of a brief psychological intervention on students' sense of belonging and educational outcomes: the role of students' migration and educational background. *J. Sch. Psychol. 75*, 41–57. doi: 10.1016/j.jsp.2019.06.002

Organisation for Economic Co-operation and Development [OECD] (2017). *PISA 2015 Results (Volume III): Students' Well-Being.* Paris: OECD Publishing, doi: 10.1787/9789264273856-en

Zaussinger, S., Unger, M., Thaler, B., Dibiasi, A., Grabher, A., Terzieva, B., et al. (2016). *Studierenden-Sozialerhebung 2015. Bericht zur sozialen Lage der Studierenden. Band 3: Tabellenband [Social Student Survey 2015. Report on the Social Situation of Students. Volume 3: Tables]*. Vienna: Institute for Advanced Studies. Available online at: https://irihs.ihs.ac.at/id/eprint/3980/1/Studierenden_Sozialerhebung_2015_Band3_Tabellenband.pdf (accessed June 10, 2021)
